# Understanding Chronic Venous Disease: A Critical Overview of Its Pathophysiology and Medical Management

**DOI:** 10.3390/jcm10153239

**Published:** 2021-07-22

**Authors:** Miguel A. Ortega, Oscar Fraile-Martínez, Cielo García-Montero, Miguel A. Álvarez-Mon, Chen Chaowen, Fernando Ruiz-Grande, Leonel Pekarek, Jorge Monserrat, Angel Asúnsolo, Natalio García-Honduvilla, Melchor Álvarez-Mon, Julia Bujan

**Affiliations:** 1 Department of Medicine and Medical Specialities, Faculty of Medicine and Health Sciences, University of Alcalá, 28801 Alcalá de Henares, Spain; miguel.angel.ortega92@gmail.com (M.A.O.); oscarfra.7@hotmail.com (O.F.-M.); cielo.gmontero@gmail.com (C.G.-M.); ccwxibanya@163.com (C.C.); leonel.pekarek@gmail.com (L.P.); jorge.monserrat@uah.es (J.M.); natalio.garcia@uah.es (N.G.-H.); mademons@gmail.com (M.Á.-M.); mjulia.bujan@uah.es (J.B.); 2 Ramón y Cajal Institute of Sanitary Research (IRYCIS), 28034 Madrid, Spain; angel.asunsolo@uah.es; 3 Cancer Registry and Pathology Department, Hospital Universitario Principe de Asturias, 28806 Alcalá de Henares, Spain; 4 Department of Surgery, Medical and Social Sciences, Faculty of Medicine and Health Sciences, University of Alcalá, 28801 Alcalá de Henares, Spain; fruizgrande@hotmail.com; 5 Department of Vascular Surgery, Príncipe de Asturias Hospital, 28801 Alcalá de Henares, Spain; 6 Department of Epidemiology and Biostatistics, Graduate School of Public Health and Health Policy, The City University of New York, New York, NY 10027, USA; 7 Immune System Diseases—Rheumatology and Internal Medicine Service, University Hospital Príncipe de Asturias, (CIBEREHD), 28806 Alcalá de Henares, Spain

**Keywords:** chronic venous disease (CVD), varicose veins, venous pathophysiology, vascular therapies, venous hypertension

## Abstract

Chronic venous disease (CVD) is a multifactorial condition affecting an important percentage of the global population. It ranges from mild clinical signs, such as telangiectasias or reticular veins, to severe manifestations, such as venous ulcerations. However, varicose veins (VVs) are the most common manifestation of CVD. The explicit mechanisms of the disease are not well-understood. It seems that genetics and a plethora of environmental agents play an important role in the development and progression of CVD. The exposure to these factors leads to altered hemodynamics of the venous system, described as ambulatory venous hypertension, therefore promoting microcirculatory changes, inflammatory responses, hypoxia, venous wall remodeling, and epigenetic variations, even with important systemic implications. Thus, a proper clinical management of patients with CVD is essential to prevent potential harms of the disease, which also entails a significant loss of the quality of life in these individuals. Hence, the aim of the present review is to collect the current knowledge of CVD, including its epidemiology, etiology, and risk factors, but emphasizing the pathophysiology and medical care of these patients, including clinical manifestations, diagnosis, and treatments. Furthermore, future directions will also be covered in this work in order to provide potential fields to explore in the context of CVD.

## 1. Introduction

Chronic venous disease (CVD) is a persistent, progressive, and frequently underestimated condition widely represented in the general population, having a huge socioeconomic, physical, and psychological impact associated [1,2]. CVD entails a broad spectrum of venous abnormalities in which blood return is seriously compromised. In the pathophysiology of CVD, the interplay between genetics and environmental factors are responsible for increasing the ambulatory venous pressure, leading to substantial changes in the whole structure and functioning of the venous system [3]. Importantly, the term CVD should be differed from chronic venous disorder, which only includes the full morphological and functional abnormalities of the venous system, but without considering the clinical manifestations and other serious concerns affecting the patient [4].

**Figure 1:**
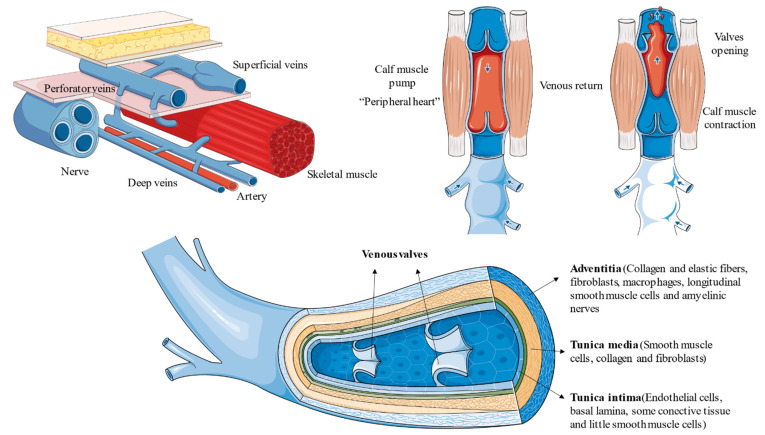
A general overview of the anatomy, physiology, and histology of venous system in the lower limbs. Superficial veins, mainly represented by the great and small saphenous veins, carry blood from the skin and subcutaneous tissues. Superficial veins might transport the blood through the saphenous junctions and the perforator veins to the deep venous system, the major contributors of the venous return. Deep veins are accompanied by an artery, nerves, and skeletal muscle at both sides, surrounded by a fascial compartment. Calf muscle pump is the most important source to assure an appropriated blood return from the lower limbs and it is frequently designed as the “peripheral heart”. Venous return is also permitted by the presence of venous valves, which are essential to prevent blood reflux. The cytoarchitecture of the vein comprises three main layers, intima, media, and adventitia, with unique properties.

Varicose veins (VVs) are the most common manifestation of CVD. Other venous signs of CVD include telangiectasias and reticular veins. Despite these, clinical signs may affect any vein in the body, the venous system located in the lower limbs is often the most vulnerable structure to suffer CVD [5]. This is mainly due to the increased resistance to overcome by the gravitational force, which is notably higher than the rest of regions in the body. In the lower limbs, three great systems may be distinguished: superficial veins, represented mainly by the great saphenous vein, the small saphenous vein, the anterior accessory saphenous vein and their tributaries; deep veins, the major transporter of blood flow; and perforating veins, connecting both systems [6]. From a histological perspective, veins are conformed by three layers: (1) tunica intima or inner layer, mainly conformed by endothelial cells (ECs); (2) the tunica media or media layer, represented by vascular smooth muscle cells (SMCs) and little elastic fibers; and (3) tunica adventitia or outer layer, which is composed by connective tissue, with an important development of elastic fibers, giving the vessel an important support and elasticity [7]. In the case of VV however, the normal structure of the vein is altered, with prominent changes in the thickness and composition of the venous wall [8]. In addition, veins present venous valves, bicuspid prolongations from the venous tissue essential to maintain the blood flow in the proper direction, impeding venous reflux [9]. Furthermore, different muscle pumps act coordinately with the venous valves to assure the unidirectional blood flow. The calf muscle, often named the “peripheral heart”, is especially considered the most prominent enhancer of venous return from lower limbs to heart [10]. Importantly, failures in both systems are also crucial to understand the pathogenesis and progression of CVD leading to a venous reflux and to venous stasis [11,12,13,14]. The anatomy of the venous system in the lower limbs, its physiology, and histology are briefly summarized in Figure 1.

The term chronic venous insufficiency (CVI) includes the most severe manifestations, such as edema, skin changes, or leg ulcers [15], frequently, associated with a poorer quality of life of these patients [16]. Different criteria are currently established to distinguish CVD presentations. CEAP (clinical–etiology–anatomy–pathophysiology) classification is a globally accepted method to study venous disorders. It is based on different medical features that permits to accurately analyze the clinical, etiological, anatomical, and pathological status of veins [17]. In this context, it is frequent to use clinical (C) manifestations to refer specific CVD presentations. For instance, VVs could be classified as C2 while CVI is comprised between C3 to C6 stages [18]. As CVD, and particularly CVI, are a worrisome and a growing concern in westernized societies while representing a challenge either for the individual or the healthcare system, the purpose of this review is to collect the current knowledge of CVD from an integrative perspective. Therefore, different approaches including epidemiology and risk factors, diagnosis, etiology, pathophysiology, and utilized treatments will be tackled. Moreover, future directions and important considerations will also be addressed.

## 2. Epidemiology and Risk Factors

CVD is a common disorder that affects an important percentage of people in the world. In general terms, the estimated prevalence of CVD ranges from 60 to 80% [19,20,21], although these data may be highly heterogeneous in the literature depending on the populations studied, the methods followed and disease definitions [22]. Most cases are defined as C0 and C1 classes, while approximately 25% of patients are diagnosed with VVs (C2). CVI (C3–C6) concern small percentage of patients with CVD, representing up to a 5% of patients [23]. The incidence of VVs was about 2.6% for females and 1.9% for males, according to the Framingham study [24]. Thus, it is recognized that the risk for suffering from VV and CVI is higher in women than men, and it also increases with age, therefore explaining its great prevalence and growing incidence [25]. Furthermore, this condition may entail important consequences for the quality of life (QOL) of the affected individuals. In fact, even patients with uncomplicated VVs note a significant reduction on their QOL and if untreated they will surely progress to CVI [26]. Only in the United States, more than 25 million people are affected by CVD and approximately 6 million present advanced stages and CVI, with an estimated cost of USD 3 billion per year [27]. Thus, it is undeniable that CVD represents one of the most frequent vascular diseases in the world, entailing an important burden not only for the individual, but also for the healthcare systems.

As above mentioned, age is one of the major risk factors of CVD. According to the Edinburgh Vein Study, ~1 in 3 patients developed CVI from the initial VV diagnosis after a 13-year follow-up, with the rate consistently augmenting with age [28]. Vuylsteke et al. [29] reported that elder individuals (>65 years) were more vulnerable to the different risk factors associated to CVD, with differential effects according to the geographical regions. Thus, elder people are notably susceptible of suffering from CVD and progress to CVI, particularly when combined with other risk factors, such as body mass index (BMI) [30] and the female gender [31]. The reasons why women are more likely to suffer from CVD, reside on different hormonal and physiological factors that make them more sensitive to develop this condition [32]. In this group, pregnant women (PW) are more prone to develop this condition, with approximately 40% of them suffering from CVD [33]. In addition, the number of pregnancies might also be a variable of note to acquire this condition. Thus, it is estimated that 20% of nulliparous women over 40 years old will undergo VVs. However, this percentage increases to 40% in the case of women who were pregnant 1 to 4 times and up to a 65% in those with five or more gestations [34]. Apart from female sex and aging, a plethora of risk factors have been identified in the development of CVD. Overweight and obesity are one of the best-established factors directly related to CEAP C categories, independently from the rest of risk factors [35]. This could be due to many plausible and synergic effects, including the pro-inflammatory status generally associated to the increased adiposity, and other factors, such as an augmented intra-abdominal pressure, which may lead to greater reflux, increased vein diameter, and venous pressure [36]. Sedentarism is another important risk factor to develop and aggravate CVD manifestations [37]. Conversely, physical activity was not related with higher risk of CVD, but it is seen as an essential method to prevent its progression [38]. Prolonged sitting and, more prominently, prolonged standing are also recognized as risk factors for suffering from CVD [39]. Frequently, both situations are related to occupational positions. Family history of CVD is also recognized as a risk factor for suffering this condition [40]. Moreover, previous events of blood clots in the veins -prominently deep venous thrombosis (DVT) has also been described as a potential risk factor for suffering from CVD [41], as well as regular smoking [42]. On the other hand, more doubts remain regarding the possible association between height and CVD. Although some studies have described a causal relationship [43], it seems that height does not influence on the progression of the disease [44]. Overall, there are many risk factors that could be related to the onset and development of CVD as they may influence different pathophysiological mechanisms involved in the onset and progression of the disease.

## 3. Clinical Manifestations

As above mentioned, CEAP classification is the most accurate and globally used method to establish a precise CVD diagnosis. It is based on clinical, etiological, anatomical, and pathophysiological criteria. Main values assigned in CEAP are summarized in Table 1. In this part we will summarize clinical manifestations (C) of CVD whereas etiology (E) and pathophysiology (P) will be subsequently discussed. The use of this system allows a better classification for patients with the aim to study their initial symptoms and progression of the disease, [45]. The main limitation of this method is, however, the interobserver variability that may depend on the experience of the physician [46]. According to CEAP, we consider C0 when there are no clinical signs of CVD, C1 with telangiectasias/reticular veins, C2 correspond to the presence of VVs, and C3–C6 as CVI, ranging from edema and dermatological alterations (C3/C4 respectively) to healed and active ulcerations (C5/C6) [18].

Clinical manifestations in the initial stages of the disease are often scarce, and the more CVD progress, the more symptomatology will appear [47]. VVs are the most representative manifestation in the venous system, although reticular veins and telangiectasias might also be recognized as important clinical signs [48]. In advanced stages, the most important expression of CVI consist of the lower limb pain, usually defined as heaviness, discomfort, or pressure exacerbated at the end of the day. Patients frequently show a malleolar edema with fovea getting worse under situations in which venous pressure increases and the lymphatic drainage is impaired [49]. Differential diagnosis should exclude other causes of the edema, such as cardiac insufficiency, hypoalbuminemia, or hypothyroidism, among others [50]. On the contrary, patients usually refer to the presence of dilated superficial tortuous veins clearly identified as an esthetic alteration. Additionally, patients with CVI are also more susceptible to suffer from DVT mainly in the femoropopliteal segment, with the added risk of pulmonary thromboembolism, a potentially mortal condition [51]. Superficial venous thrombosis (SVT) could also be distinguished as a potential clinical sign of CVD. Although for a long time this manifestation was considered as a harmless condition, recent evidence warn about the dangerous of suffering from SVT, as it is also significantly associated to an increased risk to develop DVT and pulmonary thromboembolism [52]. Moreover, in advanced stages of valvular incompetence, a superficial traumatism on large VVs could result in a hemorrhage, which could be fatal in some cases [53].

Furthermore, during advanced CVI stages (CVI ≥ 4), the inflammatory environment and edema affect skin and subcutaneous tissues, finally leading to epidermis breaking and the development of cutaneous ulcerations [54]. Prior changes in the skin includes eczema [55] and pigmentation, which appears to be caused mainly by a hemosiderin deposit occurred in early stages of CVI [56], lipodermatosclerosis, a specific chronic fibrosing panniculitis related to CVI [57] and corona phlebectatica, identified as an abnormal visible cutaneous blood vessels at the ankle with four components: “venous cups,” blue and red telangiectasias, and capillary “stasis spots” [58]. Furthermore, a rare malignant skin degeneration could also be observed in patients with CVI [59,60]. These skin changes and their clinical manifestations are strongly associated with a worse QOL of these patients [61]. Moreover, these cosmetic defects may have important consequences not only on physical but also on the psychosocial well-being of the individual [62]. It is of note that many patients with CVI may suffer from depression and anxiety, mainly due to the esthetical concerns, long-term complications and the submission to the different therapeutic regimens [63]. In this sense, Blättler et al. [64] have recently conducted a study aiming to inquire the neuropsychological impact of CVD. They observed that some individuals with mild venous pathology may refer venous symptoms that are not be correlated with their clinical state. Collectively, it can be concluded that CVD should be handled from an integrative perspective, utilizing different tools and questionnaires to accurately evaluate the QOL of these patients [65]. Particularly, Villalta-Prandoni scale (SF-36) and EuroQol 5D scale, which may be repeated over time while demonstrating its efficacy on validating this aspect of the disease [66,67]. 

## 4. Diagnosis

First, a clinical history of the patient must be carefully conducted considering allergies, previous medical prescriptions (hormonal contraceptives, anticoagulants, etc.), family history of VVs or CVD and personal antecedents of thromboembolism, cardiovascular or other relevant disease [68]. Likewise, the presence of specific symptomatology of CVD must also be assessed. Then, patients will be subjected to a physical exploration with the aim to find clinical signs of CVD, such as VVs, edema, skin changes, or venous ulcerations [69].

Regarding diagnostic methods used in CVD, color duplex ultrasound (CDU) is the most used investigational exam [70]. In fact, CDU has replaced the rest of the diagnostic techniques because of being non-invasive, reproducible, and easy to use while importing data about vein morpho-hemodynamic changes in the affected limb/s. [71]. Thus, the use of CDU is essential to conduct a proper management of the patient [72]. Similarly, the use of CDU could also be useful to assess the presence of the venous pathology in the abdominal or pelvic areas [73,74].

Likewise, the use of other procedures, such as plethysmography, might be useful to identify pathophysiological mechanisms occurring in the patient by distinguishing among venous reflux, obstruction, or both, or conversely, muscle pump dysfunction. However, it does not offer the possibility of studying anatomical features [75]. Computed tomography venography or magnetic resonance are also valuable techniques in the CVD diagnosis, although it is normally reserved to complex cases with prominent venous alterations, malformations, or abdominal affections [76].

## 5. Etiology and Pathogenesis of CVD

CVD is a vascular disorder in which venous return is compromised. According to the CEAP classification, the etiology of CVD could be as follows: (1) primary CVD; (2) secondary CVD, which in turn are divided into (i) intravenous secondary causes; (ii) extravenous secondary causes; and (3) congenital CVD [77].

### 5.1. Primary CVD

Primary CVD could be defined as a progressive process occurred in the venous wall or venous valve that leads to an abnormal dilatation and weakness in the vein, eventually resulting in pathological and demonstrated reflux. Primary CVD is frequently manifested in the saphenous veins, which is known as truncal insufficiency [78], although it could also be presented in the superficial non-truncal tributaries’ veins [79,80]. The use of animal models has shed light on the pathogenesis of primary CVD, recognizing that the sustained induction of ambulatory venous hypertension injured the vein wall and venous valves; hence, promoting the development and progression of CVD [81]. However, unlike experimental models where the increased venous pressure is induced by the researcher, the etiological agents of venous hypertension in human beings are much more complex.

Primary CVD is the result of different genetic and environmental factors. The vast majority of people with CVD are carriers of certain polymorphisms or genetic variants that are in part responsible for the development of the disease. In fact, it is estimated that the genetic component of CVD is approximately a 17% [82]. The remaining proportion resides on a wide variety of environmental components generally associated to the previous mentioned risk factors [83]. As an example, quiet standing for 30 min was enough to trigger an inflammatory response in the venous wall [84], as the venous pressure while standing is higher in comparison to the pressure while sitting and even higher than the pressure when walking. Permanent standing in the workplace starts a persistent and harming venous stasis that progressively makes subjects more vulnerable to suffer from venous reflux and CVI, particularly if combined with other factors like aging [85]. Progesterone has also been identified as an important etiological agent of CVD, as it is associated with SMC relaxation, vasodilation, and valvular incompetence [86]. Similarly, a redistribution of progesterone receptors has been shown to participate in the progression of the disease [87]. In the case of PW, significant changes occur, mainly due to hormonal, hemodynamic and mechanical factors, which induce an important remodeling in the venous system that may be associated with the onset and progression of CVD [88,89,90].

### 5.2. Secondary CVD

Secondary CVD are those cases in which the manifestation of the disease is caused by a previous event that, as mentioned below, could be subdivided into secondary intravenous—when the vein wall and valves are adversely affected—and secondary extravenous, in which there is no evidence of vein damage, but the local or systemic venous hemodynamic is impaired (e.g., external causes, such as central venous hypertension, extrinsic compression caused by a tumoral mass, by arteries such as in May-Thurner syndrome, or in case of diaphragmatic or limb muscle pump dysfunction) [77].

The most common cause of secondary intravenous CVD is a previous episode of acute DVT. DVT is a major cause of morbidity and mortality, affecting approximately 1 in 1000 of individuals per year [91]. Primary CVI is identified as a risk factor for suffering DVT [51]. In turn, clinical or subclinical DVT is identified as a potential cause of CVI [92]. A central link in this bidirectional relationship may reside on different genes shared in both pathologies, such as the THBD (thrombomodulin) and MTHFR (methylenetetrahydrofolate reductase) [43]. Apart from genetics, the increased venous pressure and damage in the venous wall may conduct to the development of both CVD and DVT. In contrast, DVT is characterized by the formation of a thrombus, caused by the interaction of the coagulation components with the endothelium and hemodynamics (stasis), generally affecting deep veins located in the lower limbs [93]. This could provoke a plethora of morphological changes in the venous wall and result in a combination of partial obstruction and reflux, leading to severe presentations, such as pain, venous claudication, edema, skin changes, and ulceration; these clinical manifestations of CVI typically characterize post-thrombotic syndrome (PTS) [94]. Moreover, secondary CVD progresses more rapid than primary CVD [95], similarly being associated with a poorer QOL of these patients [96]. It is estimated that around a 20 to 50% of patients who suffered an episode of DVT will develop PTS, while a 5 to 10% will progress to venous ulceration [97]. Thus, an adequate management of patients after being diagnosed of DVT will be essential to prevent the appearance of PTS and severe forms of CVI.

Likewise, post-traumatic CVD should also be mentioned here. The cause of vascular trauma is blunt trauma (fractures or dislocations), penetrating trauma (caused by bullets, knives, etc.), or a combination of both, and they could affect either upper or lower extremities [98]. There are five types of vascular traumas: intimal injuries, complete wall defects with pseudoaneurysms or hemorrhage; complete transections with hemorrhage or occlusion; arteriovenous fistulas; and spasms [99]. Epidemiologically, the occurrence of CVD secondary to a traumatic event in the general population is very rare (1% to 2% of the total cases), as penetrating traumas are more frequently related with this condition, with higher exposure of the military setting [98]. The clinical management of vascular traumas represent an important challenge, as they might correlate with a high morbidity and mortality. In the case of venous traumas, vascular repair and ligation are the most frequent approaches used [100]. However, previous studies have demonstrated an increased risk for developing CVI even after either ligation or repair [101]. In this line, Bhatti et al. [102] found that traumatic arteriovenous fistula was the most common reason of unusual secondary VVs; therefore, showing the impact of venous trauma on CVD etiopathogenesis. Similarly, a prior trauma is also related with a greater risk of DVT and pulmonary thromboembolism [103]. Therefore, venous trauma may represent a relevant secondary cause of CVD, being equally associated with DVT and other vascular disorders.

### 5.3. Congenital CVD

The influence of genetics in the CVD pathogenesis is extraordinarily complex [104]. As previously defined, family history of CVD is an important risk factor from suffering this condition. Notwithstanding, the genetic factors usually interact with environmental agents, the role of certain single gene mutations might be enough to cause venous disorders [105]. In this section, we summarize the association of particular genetic alterations in the development of CVD.

Venous angiodysplasias, such as Klippel-Trenaunay syndrome (KTS), are a major example of congenital CVD. KTS is a vascular disorder generally caused by somatic mutations in the PI3KCA gene [106]. Inheritance of KTS is quite complex. Some authors have considered it to be a paradominant inheritance [107], while in other cases it appears to provide a dominant inheritance [108]. Finally, inherited gene translocations have also been reported [109,110]. Howsoever, KTS is associated to an overgrowth in the vascular cells, leading to the development of different signs, such as cutaneous hemangiomas (Port-wine stains), lymphatic, or venous malformations [111]. Thus, patients with KTS exhibit a wide variety of venous complications, such as severe venous hypertension, complex reflux patterns, advanced valvular incompetence, calf muscle pump impairment and DVT, and venous ulcer development [112,113].

**Figure 2:**
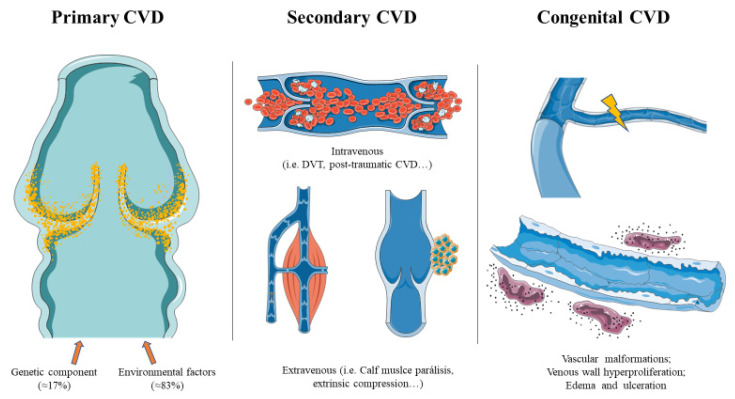
A general view on the different etiological mechanisms of CVD. Primary CVD (left) is caused by different genetic and environmental factors and it is characterized by a persistent venous hypertension responsible for the onset and progression of the disease. Secondary CVD (middle) is preceded by a pathological event, which could be intravenous (DVT) or extravenous (e.g., calf muscle dysfunction, extrinsic tumoral mass or arterial compression). Congenital CVD (right) represent a small percentage of the problem, and it is caused by single gene mutations or chromosomic aberrations.

Parkes Weber syndrome (PWS) is another congenital vascular disorder affecting capillary, lymphatic, venous and arteriovenous malformations. PWS is frequently misdiagnosed with KTS, although the latter does not present arteriovenous fistulas [114]. In addition, the gene involved in PWS appears to be RASA1, which is mutated up to a 50% of the patients. Nonetheless, 10% of the cases might be attributed to alterations in the EPHB4 gene [115]. The remaining 40% of pathogenic gene variants are still undiscovered. Similar to KTS, PWS is characterized by limb overgrowth and vascular malformations, leading to a persistent venous hypertension, dilation, and varicogenesis [116]. Moreover, approximately 1 in 4 patients with PWS show chronic venous ulcerations [117]. Thus, it would be crucial to identify and elaborate proper management of patients with either PWS or KTS, as they are more likely to suffer severe CVI and venous complications. Other syndromes related to the development of CVD include lymphedema distichiasis syndrome (LDS), characterized by FOXC2 mutations, cerebral autosomal-dominant arteriopathy with subcortical infarcts and leukoencephalopathy (CADASIL), affecting the Notch3, Chuvash Polycythemia (VHL mutations), severe congenital neutropenia type 4 (alterations in the G6PC3), and Ehlers-Danlos syndrome (EDS), with defective COL3A1 functioning [118]. Overall, these genetic mutations result, irretrievably, in the development of CVI, due to vascular and congenital malformations. Fortunately, this only represents a little group of patients with CVD. The different etiopathogenic mechanisms of CVD are presented in Figure 2.

## 6. Pathophysiology

CVD is a multifactorial disease involving complex pathophysiological mechanisms. The increased ambulatory venous pressure and vein dilation in the lower limbs promotes an intricate vascular response, leading to stasis and consequent secondary inflammatory response, and altered shear stress. Obstruction and/or reflux are the two main pathomechanisms leading to significant changes in the venous wall and pathological venous reflux, thereby contributing to the progression of CVD [119]. Moreover, these changes provide the creation of a hypoxic environment, which is thought to be an important contributor in the pathophysiology of the disease. Moreover, the role of certain gene mutations or variants are also implicated either in the etiology or pathophysiology of the disease. Similarly, epigenetic alterations have also been found in patients with CVD, as well as evidence of systemic markers of damage related to the progression of the disease [37,120,121,122]. In this section we will collect the current knowledge of these pathophysiological mechanisms occurring in CVD in order to understand the most important properties of the disease.

### 6.1. Hemodynamic and Microcirculatory Alterations

Venous return from the lower limbs entails more difficulties than arterial circulation due to the defiance of gravity. Several morphofunctional diagnostic tests are employed to investigate CVD; together with ultrasound-based instruments, plethysmograph methods are used in combination, although they have not been standardized yet. The measurements are based on the changes in volume within veins evaluating venous filling index [123,124].

In the event of CVD, venous hypertension and dilation induces a decrease in shear stress, the frictional force within ECs. ECs sense these changes and transduce the physical signals from fluid shear stress into the altered biomolecular signaling; this prompts the typical vicious cycle in CVD, inducing hypertension, venous wall remodeling, and inflammation [3,125].

In the lower leg, ankle venous ambulatory pressure normally decreases to about 35 mm Hg [126]. In patients with CVD, this value tends to increase proportionally to the CEAP stratification [127]. The different pressure gradient may be an activator of neovascularization and reflux recurrence in the event of varicose vein treatment [128]. On the other hand, continuous changes in venous pressure with edema and hypoxemia as clinical manifestations, promote microcirculatory adverse reaction and eventually leg ulcerations [129]. In the course of CVD towards CVI, the microangiopathic changes involve a decreased number of capillaries and increased permeability of these vessels and an impairment of the lymphatic microcirculation, allowing fluid, proteins, and blood cells extravasation. These changes also explain a worse skin perfusion by nutritive capillaries and the tendency of developing venous ulcers [130]. Likewise, the increased permeability triggers inflammation due to the infiltration of white blood cells (WBCs), with cytokine release, and activation of proteolytic enzymes and platelet activating factor [131]. Hence, shear stress modulating EC behavior leads to increased permeability, controlling leukocyte performance as well.

The role of venous valves should be the prevention of backflow of blood and pooling generation; however, mechanical stress promotes their incompetence. The measurement of the reflux and obstruction components in CVD patients may serve to prognose the severity of their venous clinical condition [132]. Incompetence of perforators and deep veins is highly present in severe CVI, provoking hypoxia and ulceration in the skin. Some contrasting evidence exists about the role of perforating veins in CVI onset and deterioration, questioning the need for perforator treatment [37].

Furthermore, deep venous system is surrounded by muscles that, when contracting, press veins in favor of blood return. For this reason, peripheral muscle pumps in lower limbs are other elements to analyze in order to understand the hemodynamic alterations occurred in CVD, especially calf muscle pump. Physiologically, it acts as a pump for deep leg veins within mainly gastrocnemius and soleus muscles. Sedentary lifestyle, long time standing, possible muscle/tendon/nerve/joint diseases, and being overweight promote deficient calf pump activity, leading to venous reflux, swelling, CVI, and in the worst cases, DVT. The disturbance in the calf muscle pump is then a key pathophysiological factor, which also implied a deficient healing of leg ulcers and a larger size of them in patients with profound CVI and worse prognosis [133].

Therefore, valves competence, calf muscle pumps, venous wall integrity, and elasticity determine venous pressure difference, altered hemodynamics in the lower limbs, and ultimately, chronic venous disorders or diseases [134]. Under normal conditions, in comparison to arteries, veins present a thicker adventitia layer and thinner media layer, with more content of collagen and reduced quantity of smooth muscle cells. This confers to the venous tissue the properties of reduced elasticity, but higher extensibility. Hence, blood return will particularly depend more on the accompanying muscles and valves. At present, there is a lack of studies related to venous mechanical properties as there are plenty in the case of arteries. Some reviewed literature alleges that, in the case of thrombosis, the vein gains stiffness and loses extensibility [135]. Although there is not much current evidence, comparative studies have demonstrated that the thicker venous wall in VV, adapted to undergoing increased pressure and turbulences, entails a different type of collagen composition besides a lower content of elastin (as detailed below) with decreased extensibility and elasticity contributing to the reflux effect and slackness for blood return generating pooling [136,137].

**Figure 3:**
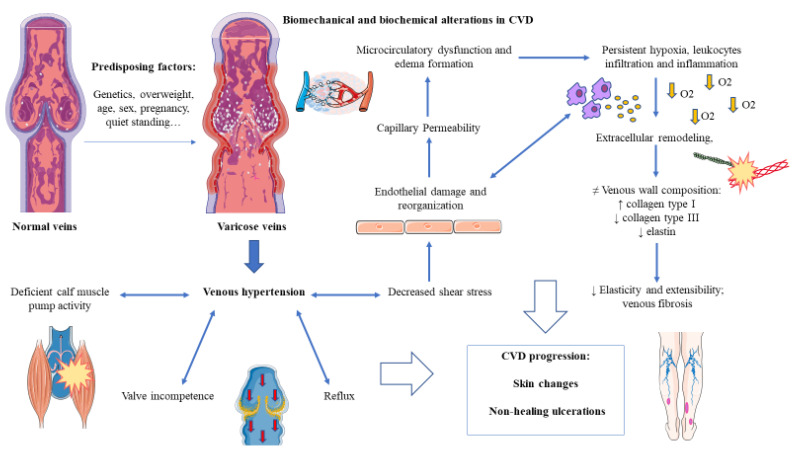
Biomechanical and biochemical alterations in CVD. As showed, ambulatory venous hypertension promotes a set of changes in the vascular wall, leading to a < n altered shear stress. The altered hemodynamic damage the endothelium, enhancing capillary permeability, leukocytes recruitment, lymphatic impairment, and edema formation. The microcirculatory dysfunction associated with these changes creates a hypoxic environment, and together with the inflammatory environment provide a extracellular matrix remodeling, eventually resulting in different venous wall compositions, reduced elasticity and extensibility, and venous fibrosis. This cycle is responsible for maintain and aggravate venous hypertension, getting worse with valve incompetence, reflux, and abnormal functioning of the calf muscle pump. Overall, these mechanisms are responsible for the progression of VVs to the most serious manifestations, such as skin changes and ulcerations.

As described, vein function is harmed by this impaired hemodynamic system, implying hemorheological changes as summarized in Figure 3. This will lead to the extracellular matrix (ECM) remodeling in adaptation, shaping vessel alterations, with important changes in composition that will later be discussed, demonstrating that biochemical alterations go together with biomechanical changes.

### 6.2. Inflammation and the Role of Endothelial Dysfunction

Inflammation is widely accepted as a hallmark of CVD, playing a pivotal role in the onset and progression of the disease [119,138,139]. Vascular inflammation is a complex process involving multiple interactions between WBCs, ECs, SMCs, and the ECM. This abnormal communication is also orchestrated by an uncontrolled release of cytokines, leading to a pathological vascular response [140]. The main causes of the exacerbated inflammatory response reside on the hemodynamic abnormalities and microcirculatory changes associated to CVD, which may initiate a persistent immune response [141]. However, fully understanding the role of inflammation in CVD is essential to highlight the role of the endothelium. Because of the hemodynamic alterations and altered shear stress during CVD, ECs might suffer important phenotypical variations. Meaningfully, these changes have been recognized as important drivers of many vascular disorders [142]. In this line, previous studies have shown a pro-inflammatory switch of ECs in people affected with CVD, therefore contributing to the entry of leukocytes in the pathological veins [143]. Herein, the role of the glycocalyx should be highlighted. During vascular diseases, the glycocalyx located in the ECs is prominently altered leading to an alteration of the endothelial mechanotransduction. This promotes the breakdown of the permeability barrier, enhancing the access of leukocytes and favoring the inflammatory reaction [144]. Thus, the proper development of CVI will lead to cumulative changes in the glycocalyx and ECs, leading to a progressive endothelial dysfunction. Accordingly, the grade of endothelial dysfunction was shown to be associated with the clinical severity of CVD [145]. Moreover, endothelial dysfunction appears to represent a central pathophysiological link between CVD and DVT [146], therefore showing the relevance of endothelial damage in the progressive inflammation and development of this condition.

In regard to immune populations, an increased infiltration of either innate or adaptative leukocytes have been evidenced in patients with CVD in comparison to healthy individuals [47,147]. Leukocytes interact with ECs in two stages: (1) a rapid phase known as I type activation characterized by EC vasoconstriction, selectin expression and von Willebrand factor release and (2) stage or “II type activation” involving adhesion molecules, cytokines, and tissue factor [148]. Monocytes/macrophages are central mediators of the inflammatory responses in CVD. Ono et al. [149] observed that incompetent venous valves were associated with an increased monocyte/macrophage infiltrate. Powell et al. [150] claimed that CVI with and without venous ulceration were related to high platelet-monocytes activation and aggregation. Furthermore, the role of macrophages appears to be particularly important in the onset and progression of CVD. Venous stasis may lead to red blood cells (RBCs) extravasation in the surrounding tissues. Then, they are degraded by interstitial macrophages and released iron from RBCs are stored in form of ferritin to later produce haemosiderin, which is responsible for the limb pigmentation in patients with CVD [151]. This altered iron deposition in the tissue might have important consequences for individuals with venous disease, inducing and sustaining the pathological oxidative stress and inflammation, while leading to the progression of CVI and leg ulcers [152,153,154]. Thus, targeting pro-inflammatory M1 macrophages induced by iron is proposed as a potential therapy in the management of CVI and venous ulcerations [155]. On the other hand, neutrophils and mast cells appear to be the first cells to interact with the affected endothelium, being important to initiate the inflammatory response in patients with CVD [156]. In the context of CVD, the abnormal activity of neutrophils appears to be related to increased adhesion molecules, lysosomal enzymes, and superoxide production, playing a key role in the development of CVI [157,158,159]. Previous studies have noticed a reduced detection of activated neutrophils in the blood of patients with VV, in comparison to healthy subjects [160]. This was explained because of a phenomenon of “leukocyte trap”, which consists of the infiltration in the tissue of leukocytes and prominently, neutrophils through tiny vessels due to venous hypertension, hypoxia, and stasis [161]. Despite this fact, an increased neutrophil/lymphocyte ratio is currently used as a marker of severity in CVD [162], thereby concluding the importance of this immune population in the pathophysiology of CVD. Conversely, the role of mast cells in the pathophysiology of CVD is unclear. Although some studies described a significant increase of mast cells in the VV wall [163], other studies did not find significant differences in mast cells populations in the wall of VV versus non-varicose [164]. Additionally, Kakkos et al. [165] reported an augmented infiltrate of mast cells in patients with family history of CVD compared to those without family history; therefore, concluding that mast cells infiltration might not be a consequence of venous hypertension. In this line, another recent study detected an infiltration of mast cells in thrombotic VV [166]. Although the role of mast cells in the pathophysiology of CVD have been evidenced, their precise implications are still undiscovered.

T lymphocytes are also crucial players of the inflammatory reaction occurring in the venous wall and valves of patients with CVD [167]. The role of T cells in vascular inflammation has been widely explored [168]. T cells could be divided into T helper (Th/T CD4+) and cytotoxic T lymphocytes (CTLs/T CD8+). Th lymphocytes orchestrate immune responses according to the threat faced. They could be differentiated into various specific subsets, such as Th1, Th2, Th17 or T regulatory (Treg) cells, among others [169]. On the other side, CTLs are involved in effector cytotoxic functions [170]. The role of these T cell subsets has been extensively studied in arterial diseases, such as atherosclerosis [171]. However, to our knowledge, there are no studies regarding particular T cells subpopulations in the pathophysiology of CVD. Nonetheless, Ojdana et al. [172] demonstrated the participation of memory Th and CTL in the pathogenesis of CVD. Furthermore, B lymphocytes were also detected in the VV in comparison to healthy veins, although its precise role remains to be elucidated [173]. Grudzińska et al. [174] defined an increase of inflammatory cytokines released by lymphocytes in incompetent great saphenous veins with reflux in comparison to non-pathological blood flow. In this line, multiple studies have demonstrated the usefulness of studying cytokine signatures of patients with CVD. For instance, Lattimer et al. [175] showed increased levels of some proinflammatory cytokines like interleukin-6 (IL-6), IL-8 and monocyte chemotactic protein 1 (MCP-1) in blood samples from patients with VV. Although an exacerbated inflammation is found in patients with CVI, Guss et al. [176] reported that, in severe cases, the level of inflammatory cytokines are diminished, thereby hypothesizing that inflammatory cytokines might be involved in tissue repair rather than damage. Similarly, Howlader and Smith [177] did not find any correlation between inflammatory markers with patient symptoms, therefore indicating the intricate role of the cytokines in CVD pathophysiology. For instance, transforming growth factor beta 1 (TGF-β1) is a central molecule involved in multiple processes of CVD. The inflammatory environment associated to CVD, induce the activation and overproduction of TGF-β1. This factor acts as a critical modulator of different targets implicated in the ECM remodeling, eventually causing vascular wall fibrosis. In turn, this exacerbates the venous wall hypoxia, ECs damage, and inflammatory reactions, leading to a greater TGF-β1 activity [178]. However, TGF-β1 signaling appears to be blocked in later stages of CVD, as previous studies reported a significant decrease in the TGF-β receptors [179,180]. Conversely, Kowalewski et al. [181] reported an increase expression of TGF-β receptor II in VVs in comparison to normal veins. Thus, TGF-β and its receptors might be relevant during the early pathogenesis of CVD, although in advanced stages of the disease, its activity might be reduced. This signaling could have important implications for future therapies, evidencing the link between inflammation and ECM remodeling in CVD.

On the whole, the role of the immune system in the pathophysiology of CVD is quite complex and it results from the pathological venous pressure and hemodynamic changes, causing and responding to valve and venous wall damage. Similarly, it reacts over the affected endothelium, which initiates the recruitment and infiltration of immune cells. In turn, the proinflammatory environment created by immune cells appears to participate in the pathogenesis of the disease, conducting to endothelial dysfunction. However, it is also unclear the possible causative role of the immunological changes, which can conversely be the outcome (and not the cause) of venous stasis in CVD patients.

### 6.3. The Hypoxic Environment

Prolonged hypoxia is crucial to understand the development and progression of vascular diseases [182]. The cause of hypoxia in CVD is a reduced oxygen supply to the vascular wall. In the case of lower limbs veins, this event relies directly on special vessels designed as vasa vasorum, mainly located in the adventitia and tunica media layers [183]. In the context of CVD, venous hypertension and blood stasis may lead to two different mechanisms of hypoxia: (1) endoluminal hypoxia, mainly due to blood stagnation that results in reduced oxygen flow detected by the endothelium and inner layers of the vein wall and (2) medial hypoxia because of the compression of vasa vasorum as a reaction of venous dilation and increased pressure, affecting the medial and outer layer [184]. At molecular levels, hypoxia leads to the activation of the hypoxia-inducible factor 1α and 2α (HIF-1α and HIF-2α). An increased activation of both transcriptional factors and their related genes in VV compared to non-VV was demonstrated; hence, demonstrating the central role of hypoxia signaling in the pathophysiology of CVD [185].

Endoluminal hypoxia may bring two distinguished responses. Firstly, acute hypoxia triggers the release of inflammatory mediators and growth factors, initiating the recruitment of immune cells. Indeed, more leukocytes can adhere to ECs when incubated under hypoxic conditions [160]. Then long exposure to hypoxia leads to the activation of HIF, upregulating the expression of key genes, such as proinflammatory cytokines, platelet-derived growth factor (PDGF), and vascular endothelial growth factor (VEGF), thereby increasing immune adhesion and recruitment in the VV [186]. Hypoxic signaling in this layer leads to intimal hyperplasia and degenerative changes in the inner wall, with a remarkable increase of the vasa vasorum as a compensatory mechanism to ensure an appropriate oxygen supply to the tunica media [187]. Nevertheless, progression of CVD entails an augmented hypoxic environment negatively affecting to SMCs. Xu et al. [188] demonstrated important phenotypical changes in SMCs from VV versus non-VV. They concluded that these cells showed increased proliferative and synthetic capacity in comparison to SMCs derived from normal veins, also being more dedifferentiated. Furthermore, these cells present a marked degeneration, losing its morphology, while increasing its phagocytic activity of either collagen or elastic fibers and even other SMCs [189]. In the same manner, due to endothelial activation, SMC migration from the medial to the inner layer has been demonstrated [190,191]. A strong cause of this abnormal behavior is the hypoxic environment. In this sense, we detected significant changes in hypoxic markers (HIF-1α, VEGF, TGF-β1, and eNOS) in cultured SMCs obtained from patients with VV under hypoxic conditions, observing a genetic and epigenetic reprogramming of these cells during CVD [192]. Moreover, in the case of sustained and long-term hypoxia, these cells showed a reduced expression of compensatory mechanisms to hypoxia, which may provoke, among other effects, an increased cellularity and hypertrophic areas, apoptosis dysregulation, and collagenization typically observed in the venous wall of patients with CVD [193,194].

Hypoxia is a major and detrimental characteristic of CVD, partly responsible for the pathophysiology in the affected veins at early, but particularly at late stages. Thus, studying oxygen supply in VV, as well as finding specific targets related to hypoxia and its signaling, appears to be an interesting and important approach in the management of CVD [184].

### 6.4. Molecular Basis of CVD: The Venous Wall Remodeling

In the venous wall, vascular cells possess different mechanoreceptors responsible for the blood flow, mainly flow-sensitive ion channels, G protein coupled receptors (GPCRs) and integrins, although the role of glycocalyx and the platelet endothelial cell adhesion molecule-1/cadherin/VEGF receptor-2 complex in the mechanotransduction process should not be discarded [195]. Thus, the blood stasis and venous hypertension occurring during CVD will activate these receptors, triggering a wide variety of molecular responses in the ECs, SMCs, or vascular fibroblasts [196,197].

Firstly, shear stress will provoke important changes in the endothelium, leading to its dysfunction, as previously described. Simultaneously, a local response to the altered environment will be developed, with remarkable changes in the different layers. In this sense, significant changes in peptide growth factors and their receptors have been identified in the venous wall of patients with CVD, including acidic fibroblast growth factor (aFGF), VEGF, and insulin-like growth factor 1 (IGF-1), and its receptor (IGF-1R) [198]. The last route appears to be particularly important in the venous wall of patients with CVD, where we have recently reported an altered homeostatic IGF-1/PAPPA/STC-2 axis [199]. TGF-β1 has also been related to the pathophysiology of CVD. Pascual et al. [200] described the important role of its dysregulation in the progression of CVD, particularly in the fibrous process of the venous wall. The effect of this factor appears to be mediated by the TGFβ receptor II, whose expression has also been upregulated in VVs, but downregulated in venous ulcers [181,201]. Conversely, Serralheiro et al. [179] denoted a decrease in the gene expression of TGF-β1 receptor II and III in advanced clinical stages of CVD. The expression of TGF-β1 is associated with the dysregulation of multiple products located in the ECM, including tissue inhibitors of metalloproteinases (TIMPs), metalloproteinases (MMPs), plasminogen activator inhibitor one (PAI-1), lysyl oxidase-like 4 (LOXL-4), fibronectin, or collagen, among others, disrupting EC and SMC behavior [178]. Importantly, this molecule and its downstream signaling appears to play a critical role in the progression of CVD, linking inflammation, vascular damage, and venous wall remodeling. However, further studies are required in order to understand the precise role of TGF-β1 in CVD. The augmented detection of these growth factors and their receptors might lead to the dysregulation of various cellular routes contributing to the pathophysiology of CVD, such as PI3K/Akt. PI3K/Akt is an important route hyperactivated under pathological conditions inducing important changes in cell proliferation, survival, motility, metabolism and growth, among other effects [202]. Ortega et al. [203] studied the role of PI3K/Akt in 110 patients with CVD according to the presence or absence of valve reflux. They found an increased activation of these components in patients with venous reflux, therefore indicating the role of this route in the progression of the disease. Similarly, the MAP kinases (MAPKs) pathway is prominently hyperactivated in the venous wall of patients with reported reflux, which may indicate the synergic effect of both pathways [204]. Interestingly, either PI3K/Akt or MAPKs were significantly higher in younger patients (<50) in comparison to elder patients, which may indicate a possible role of PI3K/Akt in the accelerated aging of the venous wall. The increased aging of the venous wall is also favored by the loss of some homeostatic mechanisms in the venous wall. In this sense, some of the changes described in the homeostatic mechanisms in CVD included an altered expression of the peroxisome proliferator-activated receptors (PPARs), associated to an abnormal lysogenesis and senescence [205] and JNK signaling, with an accelerated osteogenesis [206].

Many elements from the extensive list that conforms the pathogenesis network in CVD are Ca^2+^-dependent signaling pathways. In SMCs from varicose saphenous vein, the observation was an impaired Ca^2+^ mobilization, implying reduced contractility [207]. For its part, the RhoA/Rho kinase signaling pathway, modulates Ca^2+^ sensitization to actin cytoskeleton and ECM fibronectin assembly in SMCs, but in VVs, both have decreased activity and deposition respectively [208]. Reduced expression of RhoA is associated to abnormal EC function and morphology, which depends on EGFL7 regulation by CASZ [209]. Mentioned variants from *Nfact* family are also Ca^2+^-dependent signals for development and function of T cells [210]. In related literature search, NFATC3, NFATC4, and other isoforms of *Nfatc* family may be also relevant as all of them coordinate tidily the tissues-vessels relationship in vasculature design [211]. Moreover, it is known that intravascular pressure induces nuclear accumulation of NFATC3 isoform in SMCs in event of nitric oxide synthase activation [212], an occurrence observed in patients with CVI [122,213]. Overall, the shear stress and environmental strains induced by CVD provoke an important cellular reprogramming that eventually leads to an altered cell senescence, ECM remodeling, and other significant changes in the venous wall.

Matrix Gla protein (MGP) has a prominent role in mineralization, which is boosted in SMCs of VVs [214]. This protein is a central inhibitor of vascular calcification via carboxylation by vitamin K although the accurate mechanism is not clearly understood [215]. It is suggested that MGP must be a predictor for calcification status of VV whose modus operandi can be expressed in different ways: inhibition of calcium phosphate precipitation, formation of matrix vesicles, formation of apoptotic bodies, and transdifferentiation of vascular SMCs [216]. For its part, MGP induces TGFβ expression and parallel, induces VEGFs expression, which promotes angiogenesis, lymphangiogenesis, and vascular permeability. Some ex vivo studies have shown that by blocking TGFβ with antibodies, MGP does not have effect on VEGF upregulation [217]. As described, MGP, VEGF, cell–cell junctions and more elements depend on Ca^2+^ signaling, which is key for vascular tone and function to respond to hydrostatic pressure. It is known that the regulation of voltage dependent Ca^2+^ channels is altered with aging processes and hence affects vascular function [218]. Furthermore, some studies reported that primary VV show aberrant release of VEGF, which, in combination with other altered molecules, might increase the permeability of venous wall, allowing the infiltration of inflammation markers [219].

As reviewed above, rheological properties hold a relationship with venous wall now that there are histological differences between pathological and non-pathological veins, underlying the composition of ECM in venous remodeling. MMPs are secreted by fibroblasts and, when activated, they degrade collagen and elastin, which can affect other elements in vessel structure like SMCs migration. WBCs infiltration promotes an anomalous functioning of MMPs and TIMPs distinguishing zones with atrophy (little ECM) and hypertrophy (abundant ECM). The different levels of expression/activity of MMPs determine irregularity in VVs with dilated zones and tortuosity [220,221]. The dilation of vein due to hyperpolarization of K+ channels by MMP-2 was suggested by Raffetto et al. Since MMPs degrade collagen, peptides formed may attach to SMCs integrin receptors that entail the activation of K+ channels [222]. The changes in collagen, fibronectin, elastin, and calcium deposits establish the stiffening of vessels [223]. The deposition of certain collagen subtypes makes intimal and middle layers become thicker and, in general terms, collagen fibers increase while elastics decrease, contributing to the alteration of common orientation of SMCs [224]. For instance, type I collagen is expressed in bigger amount than III, in comparison to non-varicose veins [225]. Collagen III is involved in tissue elasticity, and the high collagen I:III ratio would also explain the low extensibility of VVs [226]. Moreover, elastin degradation, a critical event associated to an accelerated aging, generates products with cytokine-like activities, exerting chemotactic activity for leukocytes, especially polarizing towards Th1 subtypes [227]. The degradation of fibronectin is also bigger than its synthesis and deposition. This would also explain the long period of healing in typical CVD leg ulcers, now that fibronectin is synthesized by fibroblasts from skin, but the deposition is impaired [228].

Proteomics studies have elucidated the role of dermatopontin, and tenascin C, showing an increased proteolysis of these two factors in patients with VVs [229]. Firstly, dermatopontin is especially found in dermal fibroblasts, on the surface of collagen fibers, indicating a role in wound healing [230], and it increases the cellular response to TGFβ [231]. Secondly, tenascin C is an extracellular glycoprotein highly expressed in wound healing, is synthesized by SMCs in response to cell proliferation or migration [232]. Changes in both proteins could also play a vital role in the pathophysiology of CVD. Moreover, another protein that could be included in the complex network of multifactorial CVD is vinculin, a mechanosensory protein that takes part in endothelial cell-cell adhesion remodeling [233]. In any case, there is still a lack of knowledge related to these proteins and their participation in CVD pathogenesis.

Furthermore, the study of the glycome, the group of glycoconjugates united to lipids and proteins, unravels different glycosylation pathways in health and disease, becoming a prominent target for new advances towards therapy [234]. Glycosylation of ECM proteins affect to folding, solubility, binding, and degradation [235], and the accumulation of its end products increases the crosslinking of collagen. The crosslinking needs hydroxyproline amino acid to form hydrogen bonding in collagen molecules and this posttranslational modification affecting proline is highly represented in VVs, contributing to the stiffness of venous wall, aging, and inflammation.

**Figure 4:**
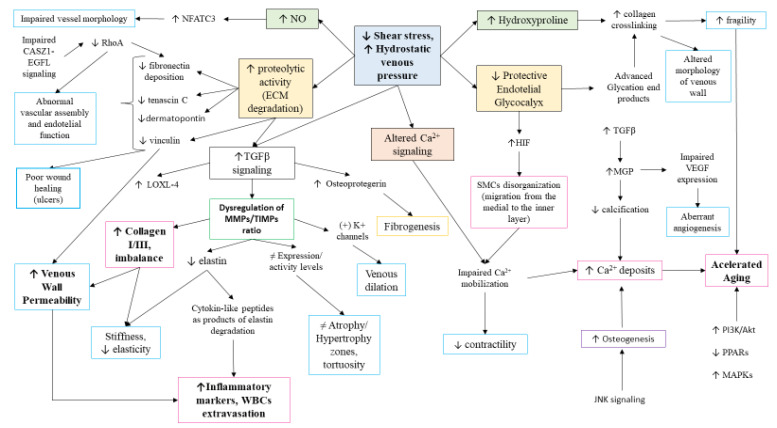
Schematic representation of molecular changes induced in venous structure resulting in different biophysical properties. As previously described altered (mostly decreased) shear stress and venous hypertension are responsible for the venous wall remodeling in VVs, influencing a plethora of molecular pathways. An increased level of hydroxyproline, reduced protective endothelial glycocalyx, altered calcium signaling, imbalance in matrix metalloproteinases and their inhibitors, enhance ECM degradation and augmented nitric oxide are the most important effects described. Collectively, these alterations are responsible for modifications in collagen and elastic fibers, venous wall permeability, disrupted vessel morphology, and functioning and multiple defects associated to the progression of CVD. Legend: Up black arrow= Increased expression; Down black arrow: Decreased expression; ≠: Differential detection.

As presented throughout the text, changes in biomechanical aspects alter the biochemical signaling pathways and these lead to changes in biophysical properties of venous wall, as summarized in Figure 4.

### 6.5. Genetics and Epigenetics Mechanisms of CVD

#### 6.5.1. Genetics

In summary, literature research shows a high number of genes related to the etiology of CVD, contributing to the pathophysiology. Many of them are intronic variants polymorphisms or single nucleotide polymorphisms (SNPs), others are mutations, and the rest are just upregulated under CVD being related to systemic alterations. A wide variety of genes are listed, being associated to alterations in blood pressure, vascular wall remodeling, and impaired wall properties (tension and elasticity), besides typical inflammation of CVD. Genome-wide association studies (GWAS) found much more variants at susceptibility loci with enough significance associated to CVD. Nevertheless, there are still few studies and peer reviewed articles related to CVD heritability and further efforts in this field are also needed.

In recent years, GWAS in VVs have allowed the identification of genetic risk factors previously unexplored. These findings showed some susceptibility SNPs for the genes of the pore-forming subunit *KCNH8* (rs727139) (the potassium voltage-gated channel subfamily H member 8) and the extracellular matrix (ECM) glycoprotein *EFEMP1* (rs17278665). *KCNH* is involved in many functions including neuronal excitability and smooth muscle contraction, which is believed to be associated to venous dilation and VV formation [236]. *EFEMP1* variants are related to MMPs and TIMPs altered expression, which leads to remodeling of ECM components and changes in vessel elasticity [237]. Another study found positive correlation between VV and polymorphisms in promoter region of *MMP-9* and *TIMP-2*, which prevent excessive ECM [238].

More genetic analyses have been related to etiology and pathophysiology of CVD in terms of hypertension and abnormal vasculogenesis, revealing as causal genes: *CASZ1*, *PIEZO1*, *PPP3R1*, *EBF1*, *STIM2*, *HFE*, *GATA2*, *NFATC2*, and *SOX9* [239]. CASZ1 (castor zinc finger 1) in its variant rs11121615 SNP has been found with strong association in severe CVD, but not in forms of this disease without VV. The transcription factor, *CASZ1*, directs the expression of EGFL7 (epidermal growth factor like-domain 7) that will lead to a signaling pathway concluding in angiogenesis stimulation and vascular assembly. A study settled that the variant rs11121615 for CASZ1 is influencing risk of developing VV [240]. For its part, *PIEZO1* is a cationic channel that functions as a sensor of shear stress by Ca^2+^ influx among ECs, which is decisive factor for vascular structure. It is also permeable to Na^+^ and K^+^, functions as a baroreceptor and is necessary for valve morphogenesis and regulation of vascular tone [241] with consequences in circulation of lymph as well [242]. A mice model showed that *Piezo1* can mediate blood flow and is followed by reorganization of ECs [243]. Loss-of-function mutations in this gene can cause autosomal recessive lymphatic dysplasia, a congenital disease that affects lymphatic system and edema in lower limbs [244]. Moreover, some variants have been associated particularly to VV recurrence, rs2911463 [43].

According to Shadrina et al., *PPP3R1*, *EBF1*, *GATA2* and *NFATC2* were genes associated to immune response or inflammation in vascular remodeling, a noted part related to VVs [239]. PPP3R1 (protein phosphatase 3 regulatory subunit B, alpha) associated variants, rs2861819 and rs2241173, have been studied as genetic determinants for CVD development, being involved in vascular integrity [33]. EBF1 (early b cell factor transcription factor 1) has a role in cell adhesion and migration during early B lymphopoiesis, being crucial for managing B cell immunity, and provides susceptibility to VV by its variant rs11135046 [245,246]. Next, the endothelial transcription factor GATA2 (GATA binding protein 2) can activate the transcription of genes involved in lymphatic vessel valve development, an impairment function correlated with lymphedema [247]. In this case, the associated variant of CVD risk found in GWAS was rs9880192 [239].

The role of NFATC2 (nuclear factor of activated T cells 2) in VV development has not been elucidated yet, but some variants have been found as well; the risk SNPs are rs3787184 and rs12625547 [246,248]. *Nfatc* signaling is calcineurin-dependent and acts in consonance with other transcription factor, FOXC2 (C2 isoform of the forkhead family transcription factor), in the regulation of GJC2, gap junction proteins of endothelial tissue. Mutations in genes FOXC2 and GJC2 are cause of disruption in venous valve function. Ulceration and hypertension will expound in absence of a proper valve control in which these genes are involved [249]. Moreover, animal models have shown that FOXC2 inactivation is correlated with abnormal shear stress sensing and junction disassembly due to cell-cell junction defects and valves impairment [250]. This problem has been studied in lymphedema-distichiasis syndrome that, as previously narrated, was associated to FOXC2 mutations.

Continuing with other possible targets, STIM2 (stromal interaction molecule 2) is an endoplasmic reticulum protein that controls Ca^2+^ concentration in cytosol and seems to be implicated in typical morbidity in aging, like autoimmune disorders [251]. GWAS studied proposed rs28558138 as risk SNPs [248]. HFE (homeostatic iron regulator) regulates iron absorption by regulating hepcidin expression. Recessive mutations confer hemochromatosis, a disorder of iron storage [252]. Iron overload is correlated with impaired endothelial function in patients with hereditary hemochromatosis [253]. On the other hand, in CVD, the RBCs extravasation and deposits of iron could highly activate MMPs or block their inhibitors, besides generating free radicals. The fact that iron could be an elicitor of venous leg ulcers has been hypothesized. In an observational study, patients with variant H63D (rs1799945) developed ulcers at an earlier age [254]. Another variant found in GWAS was rs7773004.

Next, *SOX9* or SRY-box transcription factor 9, can modulate extracellular matrix, associated with high calcium deposition and its overexpression is influenced by TGF-β1 [255]. GWAS found variant rs2241173 for varicosity susceptibility. Gene *COL2A1* (variant: rs73107980), which codes for collagen type II alpha 1 chain, component of extracellular matrix, is a target of *SOX9* whose ectopic expression had previously been linked to abnormal modeling of extracellular matrix [239,256]. Presumably, the isotype *COL1A2* has been studied in more depth, in collagen dysregulation. Genetic variations (insertion/deletion rs3917 polymorphism) seem to provide susceptibility to CVI [257].

In this extensive list, there are, finally, two genes (that were previously mentioned) that are an important link between thromboembolic disease and CVD: *THBD* and *MTHFR*. In this line, previous studies found that both conditions shared familial susceptibility [258]; hence, indicating the implication of some genetic mechanisms in the overlap of these diseases. Indeed, mutations in the two associated genes has been related to the development of either DVT or VV [43]. Blood samples obtained from VV exhibited higher levels of prothrombotic and inflammatory markers along with evidence of endothelial damage in comparison to non-VV blood [259]. These two genes could be taken part in the aberrant environment affecting the venous wall and the local response in the vein, connecting, to some extent, DVT and CVD pathophysiology. The main genes involved in the various pathophysiological mechanisms of CVD are summarized in Table 2.

#### 6.5.2. Epigenetics

The term epigenetics refers to the different molecular mechanisms involved in the modulation of the genotype into a particular phenotype. Importantly, these changes do not usually affect directly DNA sequence, although they could be mitotically and meiotically hereditable [260]. In this sense, the epigenetic mechanisms are the following: (1) DNA methylation; (2) histone modifications, including acetylation/deacetylation, methylation, phosphorylation, ribosylation, ubiquitylation, SUMOylation, and citrullination; and (3) non-coding RNA, with a central role of micro RNAs (miRNA), short-interfering RNA (siRNA), and long non-coding RNA (lncRNA) [261,262]. The epigenetic mechanisms are orchestrated by several external factors including aging, nutrition, behavior, psychological stress, physical activity, working habits, circadian rhythms, smoking, and alcohol consumption [37,263,264,265]. During cardiovascular diseases, all these components might promote an unfavorable environment that, along with the proper pathophysiological mechanisms, may lead to an epigenetic switch, thus contributing to the onset and progression of the disease [266]. In this line, shear stress represents one of the most important modulatory factors in ECs, initiating a wide variety of epigenetic responses [267]. Thus, an altered shear stress has been associated with the development of atherosclerosis [268]. In the same line, other mechanisms, such as hypoxia or inflammation, might induce the epigenetic reprogramming of the vascular cells, which in turn enhance the inflammatory and hypoxic responses [269,270].

Regarding genes regulated in CVD by epigenetic mechanisms, we will remark the role of two main pathways: DNA methylations and non-coding RNAs. DNA methylation consist of the adding of a CH3 group in the CpG sites in a process mediated by the methyltransferases enzymes [271]. DNA could be hypermethylated or hypomethylated, depending on the activity of the methyltransferases. Hypermethylation cause the silencing of the gene while hypomethylation leads to its activation [272]. Importantly, either hypermethylated or hypomethylated patterns could be detected in different loci under pathological conditions [273]. In the context of CVD, the above mentioned EBF1 and MTHFR act as critical gene modulators. EBF1 may be considered as an epigenetic regulator now that it can induce DNA demethylation, nucleosome modeling and modulate active chromatin of B cell gene networks [274]. In the case of MTHFR, previous research observed that certain polymorphic variants appear to induce DNA hypomethylation, thus leading to an abnormal expression of matrix and structural proteins, affecting DNA integrity and accelerated aging of the venous tissue [275]. Importantly, MTHFR mutations were associated with inherited hypercoagulability, as well as an increased risk of suffering from CVD and DVT [276].

In this line, transcriptomic and DNA-methylomic studies have identified microfibril associated protein 5 (MFAP5) as a gene hyperactivated in patients with VVs due to an increased hypomethylation [277]. In addition, other genes, such as ADCY3, DPEP2, HRC, PLXNB1, osteopontin, integrin β3 and CCN5 (WISP2) are importantly dysregulated in VVs, due to an altered methylation patterns also being linked to the signaling network of CVD [247,278]. However, further research is required in this field to establish the causes of these methylation disturbances.

Likewise, the role of non-coding RNAs in the pathophysiology of CVD has received more attention. Non-coding RNAs are RNAs without coding potential but playing multiple functions in biological and pathological processes [279]. In this context, miRNAs represent one of the most important non-coding RNAs, preventing protein expression acting at post-transcriptional levels. miRNAs could be upregulated or downregulated, thereby exerting their action or not in the corresponding product [280]. A single miRNA may regulate, on average, 200 transcripts and a single transcript could be regulated by different miRNAs [281]. In addition, miRNAs could be used as valuable biomarkers of disease, with important implications for diagnosis and prognosis for the patient [282]. Available studies of miRNAs in CVD are conducted mainly in the VV tissue, with important pathophysiological implications. In this sense, Jiang et al. [280] detected 14 miRNAs abnormally expressed in the VV tissue. Particularly, miR-34a, miR-155 and miR-202 appears to be the most significative components in these patients. Among the most important targets of these miRNAs were MAPKs, hyperproliferative targets, apoptotic genes, cell cycle, and p53 signaling pathways. In the same manner, Anwar et al. [283] reported altered levels of miR-642a-3p, miR-4459, miR-135a-3p, and miR-216a-5p, in the vein tissue of patients with CVD, being correlated with an altered metabolomic profile, increased proliferation and inflammation. MiR-382 appears to be a key regulator of the gene COL1A2. However, in patients with the polymorphic variant rs3917 the affinity of this miRNA with this gene product decreases, hence leading to the upregulation of COL1A2 [264]. More detailed, Zalewski et al. [284] described 31 miRNAs and 62 genes recognized as potential biomarkers of CVD. These miRNAs established a complex network leading to the upregulation of certain genes like WNK1, PRRC2B, CDS2, and the histone deacetylase HDAC5, while downregulating other targets (e.g., PABPC3). All of these interactions enhance the pathophysiological mechanisms of CVD, therefore showing the promising role of miRNAs in this condition. Respecting other non-coding RNAs like lncRNA, previous research has demonstrated the important role of these components both at physiological and pathological conditions [285]. LncRNAs similar to miRNAs are able to regulate protein expression, acting through different mechanisms either in the nucleus or cytoplasm. In the case of CVD, lncRNAs also play a vital function in the pathophysiology of the disease, influencing a plethora of genes involved in the development of CVD, mainly through metabolic, structural, and inflammatory mechanisms, among others [286,287]. Despite the relevance of non-coding RNAs in the pathogenesis of CVD further studies are needed to deepen on the pathological role of these elements. Moreover, to our knowledge there are no studies regarding the use of non-coding miRNAs as diagnostic or prognostic factors of CVD, and future efforts could be placed on this direction.

Finally, it is important to highlight that epigenetic mechanisms are reversible, as well as contribute to the development and progression of CVD, it might act favorably by modulating certain factors, such as diet, physical activity, emotional management, and rest while limiting alcohol consumption and tobacco smoking.

### 6.6. Systemic Affections

There is a growing body of evidence indicating that CVD is not only a local disease, but also a systemic malady, with detrimental implications for the whole organism. Contrary to popular beliefs and as reviewed throughout the study, the presence of VV is much more than an esthetic concern, but also a worrisome condition with many symptoms and signs, entailing devastating consequences in the QOL of the patients [2]. In this sense, it is known that individuals with CVD present a disrupted serum levels of many compounds including proinflammatory cytokines [176], circulating parameters (prominently estradiol, homocysteine, and VEGF) [288], oxidative stress indicators [122] and epigenetic markers [285]. In addition, much of these agents increase at advanced stages of CVD, therefore suggesting the systemic implications of the disease.

Moreover, these effects could be even greater in the most vulnerable populations, like PW. As above mentioned, this group is particularly susceptible to suffer from CVD. In this sense, we conducted multiple studies regarding the impact of this condition during gestation in comparison to healthy pregnancies. The placenta is a crucial organ during pregnancy, involved in multiple functions, providing oxygen and blood supply to the fetus, among other functions [289]. Thus, a proper working of the placenta is key to determine pregnancy success and fetal well-being. Conversely, impairment in the placental function and abnormalities on its structure during pregnancy could enhance fetal reprogramming, which may negatively impact, even in adulthood [290,291]. In this context, we have reported significant changes in the placentas of women with CVD, showing increased damage and hypoxia [292], villous calcifications [293], altered lipidomic profile [294], augmented angiogenesis and lymphangiogenesis [295], ECM remodeling [296], and evidence of oxidative damage, in either the mother or fetus [297]. Importantly, many of these changes have also been reported in the placentas of patients with pre-eclampsia, a hypertensive disorder affecting the arterial system [298]. Although contrary to gestational venous hypertension, pre-eclampsia could be a severe and life-threatening condition, our results show the CVD course, with significant changes in the placenta of PW affected with this condition, and the necessity of further studies to assess the impact of the disease, in both fetal and maternal well-being.

## 7. Therapeutical Approaches in CVD

The medical care of CVD entails different strategies that can be used either alone or in combination, in order to maximize the clinical management of the symptomatology, prognosis and therefore the QOL of patients. In this sense, three main approaches are worth mentioning here: (a) compression therapies, targeting venous hemodynamics; (b) medical interventions directed to control venous insufficiency, and (c) pharmacological therapies directed to specific pathophysiological mechanisms of the disease [299]. It is of note that elder people and patients with additional comorbidities, despite requiring more interventions, are good responders to the different therapies received as younger patients or patients without any affection [300]. Thus, the entire population affected by CVD might be beneficiary from available therapies.

Compression therapy act by increasing the interstitial pressure, thus decreasing both the superficial and the deep veins caliber, reducing venous pressure and edema while promoting the contractile activity of the calf muscles [301]. Compression stockings are easy to use, and frequently, they are the first conservative measure to take, achieving significant improvements in most patients without causing much discomfort [302]. Thigh length stockings provide the greatest efficacy regarding volume reduction and venous hemodynamic, without affecting patient comfort [303]. In addition, compression stockings favor the healing of ulcers associated to CVI; hence, decreasing the recurrence of ulcerations in venous diseases after surgical intervention [304]. However, the usefulness of compression therapy after VV treatment has a limited degree of evidence [305,306]. Even so, it must be considered that patients with peripheral arterial disease require a careful application of this particular treatment, as it might interfere with blood circulation in the lower limb and worsen the underlying disease. Therefore, the ankle-brachial index should be calculated to discard arterial involvement, which would contraindicate the use of compression stockings in specific patients [307].

Similarly, CVD can be treated pharmacologically through various venoactive agents. These compounds aim to decrease vascular permeability, ameliorating the inflammatory response and, to some extent, they may increase vascular tone and operate on platelet aggregation [47]. Pharmacological treatments range from saponins, flavonoids, pentoxifylline, micronized purified fractions of flavonoids, and acetylsalicylic acid [308]. The main applications of these pharmacological agents are guided to treat non-serious symptoms like pain or edema at initial stages. In addition, a moderate level of evidence also supports their use in some clinical signs of CVI including trophic disorders, cramps, restless legs, swelling, and paresthesia, but its efficacy in venous ulcerations is inconsistent [309]. Nevertheless, certain drugs, such as sulodexide or purified flavonoids, appears to play a promising role in the management of CVI and venous ulcers, particularly when combined with compression therapy [310,311]. In the event of patients with refractory ulcers, acetylsalicylic acid could be utilized under some cautions [14,312]. However, some authors question the usefulness of the pharmacological therapy in the management of patients with CVD, claiming for further studies to unravel the real position of these drugs [313]. Anticoagulants play a specific role in a few venous diseases, such as DVT, SVT, PE, and they have been proposed in other cases of CVD, such as PTS and venous ulcerations [37].

It should be emphasized that different technologies and surgical approaches have been validated as interventional treatments in the management of CVD. The rationale of this type of treatment relies on the effective control, either of the localized progression at early stages of the disease, or on the prevention of long-term complications, also targeting optimal cosmetic results [314].

Varicose vein treatment is the most practiced interventional approach in CVD patients. Ultrasound-guided sclerotherapy is one of the best-known procedures in the management of VV of the lower limbs. Sclerotherapy is based on the use of different chemical agents (polidocanol, sodium tetradecyl sulfate, glycerin, etc.) to ablate veins, venules, and in general, any type of varicose manifestation in patients with CVD [315]. This therapy is indicated especially, but not only, in patients with baseline comorbidities, where more aggressive techniques could be contraindicated [316]. Moreover, sclerotherapy can be an alternative to other procedures in patients with advanced CVI and saphenous vein incompetence who are not candidates for surgery or endovenous techniques [317]. The results of sclerotherapy are similar to other interventional techniques, properly managing the associated symptoms of CVD with a low rate of adverse effects [318]. Moreover, sclerotherapy has provided substantial benefits for patients with superficial venous reflux, enhancing ulcer healing and preventing recurrences in similar rates than other procedures [319]. More recently, sclerosant foam-based and catheter-based procedures have proven to achieve outcomes in the range of thermal ablation techniques at mid-term [37]. However, most trials show similar results for all VV therapies, in regard to patient-reported outcomes. 

As to open surgery (saphenous junction ligation and stripping) in VV disease, conflicting data exist [37] in terms of mid-long term recurrence; some literature data report on a superior efficacy to medical and conservative approaches to resolve the symptoms associated with VV [320,321]. Before the introduction of the endovenous thermal or chemical ablation interventions, surgery was considered the gold standard for the treatment of CVI for a long time [322]. However, because of its invasiveness, the higher rate of potential complications, including the appearance of hematomas, infections on the surgical wound or nerve injury, the surgical approach is usually reserved to patients with large and tortuous dilations in superficial VVs or patients with some vascular malformations [323].Because of that, nowadays, ultrasound-guided endovenous ablation has replaced previous techniques used in the field of CVD. Two different types of endovenous thermal ablation can be distinguished: endovenous laser ablation (EVLA) and radiofrequency ablation (RFA). Both techniques are based on the induction of a controlled thermal injury at the VV level, leading to a thrombotic occlusion eventually causing fibrosis of the saphenous vein [324,325]. This technique has now replaced surgical intervention because it offers similar results with less convalescence and a very low rate of complications [326,327]. Serious complications are rare and include DVT and SVT, pigmentary changes, nerve damage.

More recently cyanoacrylate-based options have been validated as alternative techniques in VV treatment, though the cost/effectiveness ratio may be unfavorable in many cases [37].

Finally, there are certain therapeutic alternatives reserved for deep vein occlusions/obstructions in acute DVT or in PTS, as it is the case of the endovenous PTA + stent. The use of stent in patients with CVD is indicated in presence of chronic iliac vein occlusions which are non-responders to medical interventions [328]. Conclusively, as presented in Table 3, we can observe that CVD has an amalgam of possible therapeutic approaches, which may be used to improve or alleviate the clinical manifestations of this debilitating disease.

## 8. Future Directions in CVD—Towards Clinical and Translational Improvements

CVD is a multifactorial disease that generates a huge economic burden, besides the impact on patient QOL, demanding extensive treatment and sometimes hospitalization. Given the rising prevalence, these days CVD is still an underestimated and underdiagnosed malady. For this reason, scientific evidence and clinical management must fulfill the duty of achieving enough knowledge to engender practical guidelines, in order to restore normalcy for these patients and diminish healthcare costs.

Medical technologies are supporting the optimization of current treatments. One of the most used, foam sclerotherapy, may benefit patients for some 5 years [329], though a higher success rate is reported with complementary techniques, such as catheter foam sclerotherapy with tumescence and irrigation [37]. For its part, tissue engineering is facing approaches in valve development for replacement, finding adequate materials to reach desired outcomes [330]. In the diagnosis area, new ultrasound technologies are more sophisticated and effective, enabling to reach better visualizations of blood flow, and diameter and compressibility in veins, which could provide an earlier diagnostic [331]. Moreover, further molecular research is required for a better understanding of CVD pathogenesis, especially in the field of epigenetics, which would allow to treat each case individually. Precision medicine based on miRNAs and DNA changes could be used as prognostic biomarkers and for earlier diagnosis too.

Nevertheless, we ought to considerate that prevention is better than cure. The burden of inheritance in the CVD represents only ~17%, which means that the remaining 83% can be modulated in order to avoid its manifestation. In this line, nutrition intervention and physical exercise programs are the two most cost-effective feasible lifestyle factors to promote health and independence. Both hold enough evidence to verify their ability to reduce oxidative stress and pro-inflammatory markers.

It is undeniable that the rising results of interventional studies related to adherence to a Mediterranean diet demonstrate the cardioprotective effects and anti-inflammatory properties on individual’s health. A well-balanced low-carb diet with plenty of fiber, vitamins, polyphenols, and polyunsaturated fatty acids (PUFA) satisfy the well-functioning of the immune system. On the contrary, Westernized diets denote a lack of micronutrients that lead to malnourishment and impaired immune competence associated to the origin of chronic diseases, such as CVD, obesity, cardiovascular, type 2 diabetes, or some types of cancer [332]. Indeed, previous studies have demonstrated that old adults with chronic venous leg ulcers present some worrisome markers of malnutrition, with high plasma levels of n-6/n-3 PUFA ratio, and low consumption of vitamins and fiber, accompanied by high saturated fat and sugar consumption [333]. There is a need to include dietary and lifestyle interventions in medical guidelines, which would improve the success of pharmacological treatment as well [37].

Furthermore, in order to reduce symptoms of CVD, some physical exercise trials have already been performed. Physical conditioning of CVD patients has defied the impaired calf muscle pump to handle patient disability and decreased exercise capacity [334], showing improved muscle hemodynamics and function [335]. A six-month program of structured exercise in patients with CEAP 4, 5, or 6 was demonstrated to improve calf muscle pump strength [336]. With the aid of compression therapy, exercise has also shown promising results in microvascular changes in the process of venous leg ulcer healing in CVI patients [337]. In any case, a more comprehensive and integrated approach should be advocated to target a series of epigenetic factors, which undoubtedly affect CVD onset and course.

Additionally, age represents one of the main risk factors to suffer from CVD. Life expectancy has significantly increased; however, quantity does not seem to imply QOL now that the rising morbidities are a threat to society and the economy as well [338]. Longevity and physical status, conditioned by obesity and sedentariness, are the main risk factors for the increasing incidence of CVI in elderly [37,339]. Malnutrition is also a common condition in the elderly, associated with sarcopenia and frailty, and these correlate with higher rates of mortality and longer hospital stays. Evidence alleges that strategies, such as routine screening of nutritional status, would provide, again, earlier diagnostics [340]. Nevertheless, sooner nutrition care, routinely, and lifestyle modifications would ameliorate prognosis in cases of age-related diseases [341]. This is one of the hallmarks in science targeting healthy aging.

In the same manner, due to overweight conditions and inflammatory status—pregnant women are another group to carefully watch. The existing possibility of developing CVI during pregnancy implies placental damage induced by oxidative stress [297] and the impact of this stress on epigenetic patterns can be determinant for the occurrence of vascular diseases in the offspring, even in adulthood [342]. Nutritional intervention during this period, together with tailored physical exercise may help to reprogram epigenetic mechanisms for the health status of the mother and future newborn.

**Figure 5:**
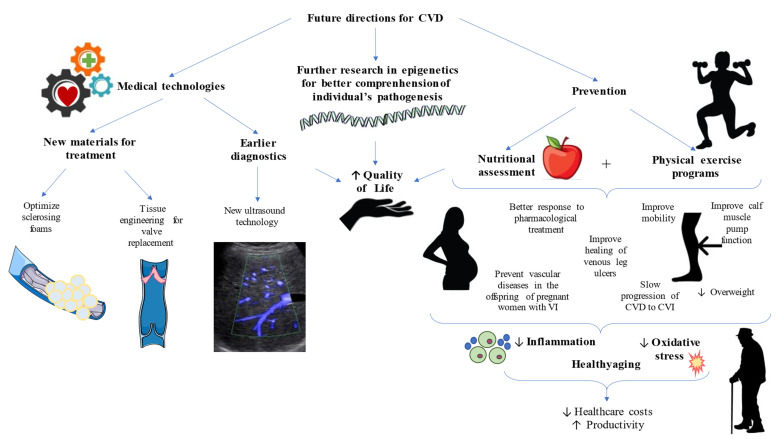
Future directions for CVD. The emergence of novel therapeutic or diagnostic methods are playing a key role in the clinical management of the patients with CVD. However, it is equally important to “deep” on the epigenetic and individual mechanisms, to allow for better comprehension of the disease, opening the possibilities of novel translational approaches. Perhaps one of the most disregarded and important factors are encompassed around prevention. In this sense, nutritional assessment and physical exercise could be powerful tools in the handle of patients affected by CVD, as they might favorably regulate clinical signs, pathophysiological mechanisms, such as inflammation or oxidative stress, and even psychological variables. Overall, these points will be crucial to improve the QOL of the patients, which might be particularly vulnerable by other variables (e.g., pregnant women or elder people).

We should also note that chronic diseases, such as CVD, implying overweight, pain, and morbidities impeding self-independence, are causes of high prevalence of depression and other mood disorders [63]. These reasons, together with psychological affections and disturbed QOL, entail a high number of medical sick leaves. It would be fruitful to remind the population about the importance of self-application of good habits, encouraging them from childhood. In this context, investment in prevention would play a prominent role, for instance, incorporating programs of adequate nutrition, psychological support, and physical exercise in education, and in medical assessment. These future directions, herein discussed, are summarized in Figure 5.

## 9. Conclusions

CVD is a progressive and disabling condition widely represented in the global population. Prolonged exposure to genetic and environmental risk factors may lead to important biophysical and biochemical changes in the venous system, entailing a complex vascular response. A growing number of studies, focusing on CVD, evidence the relevance of this vascular pathology, prominently in most advanced stages (CVI) [1,2,3]. Herein, we collected some of the most relevant data on such an intricate topic, with special emphasis on the pathophysiological and medical perceptions of the disease. Additional studies are needed in order to obtain further understanding of CVD, exploring novel translational and medical approaches to improve the QOL of these patients. Finally, society should be aware of the real impact of the disease, and attempt to adopt measures to prevent the development and/or progression of CVD to CVI (e.g., by increasing physical activity or through nutrition and lifestyle interventions). An integrative perspective of this condition would bring immediate benefits to the clinical management of these patients, particularly for the most vulnerable groups, such as elder people, individuals with extra comorbidities, and pregnant women.

## Figures and Tables

**Table 1:** Last revision of CEAP classification on CVD.

Clinical (C) Classification	Etiologic (E) Classification	Anatomic (A) Classification	Pathophysiologic (P) Classification
C0 No visible or palpable signs of venous disease C1 Telangiectasias or reticular veins C2 Varicose veins C2r Recurrent varicose veins C3 Edema C4 Changes in skin and subcutaneous tissue secondary to CVD C4a Pigmentation or eczema C4b Lipodermatosclerosis or atrophie blanche C4c Corona phlebectatica C5 Healed C6 Active venous ulcer C6r Recurrent active venous ulcer	Ep Primary Es Secondary Esi Secondary—intravenous Ese Secondary—extravenous Ec Congenital En No cause identified	As Superficial Old New Description 1. Tel Telangiectasia 1. Ret Reticular veins 2. GSVa Great saphenous vein above knee 3. GSVb Great saphenous vein below knee 4. SSV Small saphenous vein AASV Anterior accessory saphenous vein 5. NSV Nonsaphenous vein Ad Deep 6. IVC Inferior vena cava 7. CIV Common iliac vein 8. IIV Internal iliac vein 9. EIV External iliac vein 10. PELV Pelvic veins 11. CFV Common femoral vein 12. DFV Deep femoral vein 13. FV Femoral vein 14. POPV Popliteal vein 15. TIBV Crural (tibial) vein 15. PRV Peroneal vein 15. ATV Anterior tibial vein 15. PTV Posterior tibial vein 16. MUSV Muscular veins 16. GAV Gastrocnemius vein 16. SOV Soleal vein Ap Perforator 17. TPV Thigh perforator vein 18. CPV Calf perforator vein An No venous anatomic location identified	Pr Reflux Po Obstruction Pr,o Reflux and obstruction Pn No pathophysiology identified

CEAP: clinical–etiology–anatomy–pathophysiology; CVD: Chronic venous disease.

**Table 2:** Principal causal genes associated to CVD. ECM = Extracellular matrix, VV = Varicose veins.

Gene	Whole Name	Chromosome and Location	Variant(s) (rs ID)	Risk Allele	Mutation	Highlighted Associated Function	(Possible) Implication in CVD Etiology/Pathogenesis	References
KCNH8	Potassium voltage-gated channel subfamily H member 8	chr3:19293203 (3p24.3)	rs727139	A > G	-	Smooth muscle contraction	Venous dilation and VV formation	[237]
EFEMP1	EGF containing fibulin extracellular matrix protein 1	chr2:55868859	rs17278665	C > G	-	Cell adhesion and migration	Remodeling of ECM components and changes in vessel elasticity by altering the expression of MMPs and TIMPs	[238]
MMP-9	Matrix Metallopeptidase 9	chr20q11.2-q13.1	-	-	✓	ECM degradation	Collagen type I degradation entailing stiffness of vein	[239]
TIMP-2	Tissue inhibitor of metalloproteinases 2	chr17q25.3.	-	-	✓	Inhibition of MMPs	Lower expression implies higher collagen degradation by MMPs	[239]
CASZ1	Castor zinc finger 1	chr1:10765520 Intron 1	rs11121615	C > T	-	Transcription factor for EGFLD7	Angiogenesis stimulation and aberrant vascular assembly	[241]
PIEZO1	Piezo type mechanosensitive ion channel component 1	chr16:88769137 (16q24.3)	rs2911463	G > A/G > C/G > T	✓	Shear stress sensing by Ca^2+^	Impaired function implies aberrant vascular structure, ECs reorganization and edema	[242,243,244,245]
PPP3R1	Protein phosphatase 3 regulatory subunit B, alpha	chr 2:68262089 (2p14) Intergenic	rs2861819	G > A/G > C	-	Ca^2+^ sensitivity	Abnormal vascular integrity	[43]
EBF1	Early B Cell Factor Transcription Factor 1	chr5:158803005	rs11135046	G > A/G > T	-	Adhesion and migration in early B lymphopoiesis	Possible epigenetic reprogramming and B cells activation	[247,248]
GATA2	GATA Binding Protein 2	chr3:128578726	rs9880192	G > A/G > C	-	Lymphatic vessel valve development	Impaired function implies lymphedema	[240,249]
NFATC2	Nuclear Factor of Activated T cells 2	chr20:51541298 chr20:51538108	rs3787184 and rs12625547	A > G T > G	-	Induces immune response or inflammation in vascular remodeling	Not well understood; Acting in consonance with FOXC2 and GJC2.	[248,250]
FOXC2	Fork-head box protein C2	16q24	-	-	✓	Critical product in developmental processes	Inactivation implies abnormal shear stress sensing and valve incompetence	[251,252]
GJC2	Gap junction gamma-2	1q41-q42	-	-	✓	Implicated in the gap junctions between cells	Cell-cell junction defects and valve incompetence	[251]
STIM2	Stromal interaction molecule 2	4:26857601-27025381 (4p15.2) Intergenic variant Mapped gene(s): STIM2, TBC1D19	rs28558138	G > C	-	Controls Ca^2+^ concentration in cytosol	Higher Ca^2+^ deposition	[250,253]
HFE	Homeostatic iron regulator	chr6:26090951 chr6:26267527	rs1799945 and rs7773004	C > G C > T A > C/A > G/A > T	✓	Regulates hepcidin expression, involved in iron storage	Iron overload implies endothelial dysfunction. Moreover, activation of MMPs and inhibition of TIMPs, deposits of iron, RBCs extravasation	[254,255,256]
SOX9	SRY-Box Transcription Factor 9	17:72032304 17q24.3	rs2241173	A > C A > G	-	ECM remodeling	Influenced by TGF-β1may involve higher Ca^2+^ deposition	[24,257,258]
COL2A1	Collagen type II alpha 1 chain	12:47793818 (12q13.11)	rs73107980	C > G C > T	-	Coding collagen type II alpha 1	Abnormal modelling of ECM	[239]
COL1A2	Collagen type I alpha 2 chain	chr7:94431047-94431048	rs3917	(indels)	-	Coding collagen type I alpha 2	Collagen dysregulation, higher susceptibility to CVI	[259]
THBD	Thrombomodulin	20p11.21	-	-	✓	Related with thromboembolic diseases	Prothrombotic markers	[43]
MTHFR	Methylenetetrahydrofolate reductase	1p36.22	-	-	✓	Related with thromboembolic diseases	Prothrombotic markers	[43]

**Table 3:** Medical therapies, uses, and level of evidence of current available treatments in patients with CVD.

Treatment	Uses	Level of Evidence and Recommendation
Compression therapy (stockings, bandages, adjustable compression wraps)	Initial therapeutical method of CVD A powerful tool in CVI ulceration Increase the efficacy after interventional procedures	IB [303] IB [305] IA [307,308]
Pharmacological therapies	Venotonics to ameliorate early symptoms (Pain, edema) Flavonoids and derivates as complement therapy with compression stockings in venous ulcers	IIaA [310] IIaA [311,316]
Sclerotherapy	Second-line treatment for patients who are not candidates for endovenous ablation or surgery First-line therapy in patients with recurrent CVD and fragile patients with venous ulceration	IA [319] IIaB [320]
Endovenous thermal ablation	First line therapy for patients with CVD and great saphenous vein reflux	IA [318,328]

## Data Availability

Not applicable.
